# The Past and Present of an Estuarine-Resident Fish, the “Four-Eyed Fish” *Anableps anableps* (Cyprinodontiformes, Anablepidae), Revealed by mtDNA Sequences

**DOI:** 10.1371/journal.pone.0101727

**Published:** 2014-07-08

**Authors:** Luciana Almeida Watanabe, Marcelo Vallinoto, Nils Asp Neto, Janice Muriel-Cunha, Ulrich Saint-Paul, Horacio Schneider, Iracilda Sampaio

**Affiliations:** 1 Laboratório de Filogenômica e Bioinformática, Instituto de Estudos Costeiros, Universidade Federal do Para (UFPA), Campus de Bragança, Brazil; 2 Laboratório de Geologia Costeira, Instituto de Estudos Costeiros, Universidade Federal do Para (UFPA), Campus de Bragança, Brazil; 3 Center for Marine Tropical Ecology, University of Bremen, Bremen, Germany; Bangor University, United Kingdom

## Abstract

Historical events, such as changes in sea level during the Pleistocene glacial cycles, had a strong impact on coastal habitats, limiting connectivity and promoting the genetic divergence of various species. In this study, we evaluated the influence of climate oscillations and the possibility of estuary function as a barrier to gene flow among populations of the four-eyed fish, *Anableps anableps*. This species is fully estuarine-resident, has internal fertilization, is viviparous and does not migrate across long distances. These features make the four-eyed fish an excellent model for the study of evolutionary processes related to genetic differentiation of species and populations in estuaries. The evolutionary history of *A. anableps* was inferred from phylogeographic and population analyses using sequences of the mitochondrial DNA Control Region of 13 populations distributed in the Amazon and Northeast Coast of Brazil from Calcoene (Amapa) to Parnaiba (Piaui). The 83 retrieved haplotypes show a pattern of four distinct mitochondrial lineages, with up to 3.4% nucleotide divergence among them. The evolutionary reconstruction suggests that these lineages diverged recently in the late Pleistocene/early Holocene after the Atlantic Ocean reaching current levels. Analysis of variability, neutrality and the genetic expansion pattern revealed that the lineages have distinct characteristics, which were shaped by the different geomorphological features of coastal regions combined with sea level oscillations over a very long period of time. Only few neighboring populations show a discreet gene flow. This study may also be helpful for designing new experiments to better understand the geomorphological evolutionary history of the estuaries of the Amazon and the Northeast Coast of Brazil using estuarine-resident species as a model.

## Introduction

Organisms that inhabit coastal waters such as estuaries or lagoons may show more genetic differentiation than strictly marine species due to the discontinuity of these natural ecosystems and the relative isolation of the great ocean currents [1]. Estuaries are important transition zones between freshwater and marine environments [2] and are ephemeral in geological terms [3]; they have complex dynamics with daily and seasonal changes in salinity, turbidity and temperature. Furthermore, estuaries represent an environment of high productivity and are considered natural nurseries for many marine and estuarine species [4], [5].

Historical events, such as changes in the sea level during the Pleistocene glacial cycles, may have exerted a strong impact on coastal habitats by limiting connectivity and promoting genetic divergence between species [6]–[9]. During the Pleistocene, there was an alternation between glacial and interglacial periods [10]. In the last glacial period, the sea level dropped approximately 130 m below the current level, leaving the continental shelf completely exposed [11]. In the Holocene, the current interglacial period that began approximately 10,000 years ago, the sea level began to rise, creating the current estuaries approximately 5100 years ago [3].

The fluctuations in sea level in Brazil caused changes in coastal and estuarine regions, mainly in the Amazon coast. The Amazon River Delta is located in this region, which has the largest discharge of freshwater in the world [12] and could potentially serve as a barrier to the dispersal of species. The coastal regions of both Amapa and Para states have different geomorphological features [13]–[15]. The coast of Amapa is classified into two regions, Estuarine (nearest the mouth of the Amazon), and Oceanic (further north, encompassing the Amapa and Calcoene estuaries)[16]. For Para and Maranhão states, the authors have described five different geomorphological sectors: the Para Platform, Caete Basin, Gurupi Bay, Turiaçu Bay and Cumã Bay [17].

In the Para Platform (estuaries 4 to 7), great cliffs are maintained by sediment from the Pirabas/Barreiras formation, which acts as barriers to the external action of waves and tidal currents. In this area, the estuaries are narrow, with a length of only two kilometers. The estuarine channels may reach up to 60 km on the mainland and have no interconnections between adjacent estuaries [15]. Conversely, the Caete Basin (estuaries 8 to 11) is characterized by the presence of inactive cliffs with widely distributed estuaries reaching up to 30 km in length. The estuarine channels in this region may extend up to 100 km towards the continent and, unlike the Para Platform, the estuaries here are interconnected [15], which also appears to be the case for the other estuaries from Maranhao to the Parnaiba Delta.

Several studies indicate that marine environments can operate as barriers inhibiting gene flow between organisms that spend their entire life cycle in estuary ecosystems, as is the case for the four-eyed fish, *Anableps anableps* Linnaeus, 1758. This species belongs to the family Anablepidae and is considered an estuarine-resident species [18].

*A. anableps* has attracted the attention of many researchers because its retina is split horizontally, allowing for an aerial view and an aquatic view simultaneously [19], [20]. It is an epipelagic fish that can adapt only to a narrow range of salinity (stenohaline), and it is viviparous with internal fertilization [21]. The distribution of *A. anableps* extends from the Gulf of Paria in Venezuela to the Parnaiba Delta in the state of Piaui, Brazil. Moreover, the *Anableps* genus has two other species: *A. microlepis* Müller & Troschel, 1844, which is considered the sister species of *A. anableps* with whom it lives in sympatry in South America, and *A. dowie* Gill, 1861, which occurs in the Pacific (Central America) and is considered the most primitive species of the genus [22].

The life history of *A. anableps* shows that this fish has a low potential for dispersal, mainly because it does not have a period of planktonic larval dispersal (PLD) and is stenohaline and resides in estuarine regions throughout its life cycle; therefore, phylogeographic studies are of great importance in uncovering the role of historical events in the diversification of this species. Nevertheless, *A. anableps* is an excellent target for phylogeographic studies due to its previously described biological and ecological characteristics. In this context, the phylogeographic study of *A. anableps* will be of great importance for understanding the patterns of genetic variability, gene flow and levels of connectivity among populations in the estuaries of the Amazon and Northeast Brazil. Coalescence analyses may also shed light on how the evolutionary history of the estuaries correlates with the sea level alterations that occurred during the Pleistocene and the Holocene.

## Results

### Genetic diversity

Comparison among the 393 sequences obtained in the present work showed 70 variable sites (52 parsimony informative and 18 singleton variable sites) that defined 83 haplotypes. Forty-two haplotypes (43%) are represented by a single sequence in the whole sample, which are mostly concentrated in the Para Platform. The haplotype diversity varied from 0 in the Parnaiba estuary to 0.933 in the Amazonas river estuary.

Nine haplotypes were detected for the two populations of the Amapa state coast: haplotype 1, which is the most frequent, is observed in the Calcoene and Amapa estuaries, which also share haplotype 3. Haplotype 2 was observed only in Calcoene estuary while haplotypes 4 to 9 occur only in specimens of the Amapa river estuary. The populations from the Amazonas river mouth to the Maracana estuary (3 to 6) do not share haplotypes as shown in Table 1. Haplotype 43 is widely distributed, occurring in one population from the Para Platform (Urindeua), in all populations from the Caete Basin, and also in populations from Maranhao and Parnaiba Delta. Curiously, haplotype 43 is the only haplotype present in fish of the Parnaiba Delta (Table 1).

**Table 1:** Haplotype distribution by estuary.

Haplotypes	No.	Estuaries												
		1	2	3	4	5	6	7	8	9	10	11	12	13
1	36	+	+											
2	1	+												
3	2	+	+											
4 to 9	12		+											
10 to 16	10			+										
17 to 19	17				+									
20 to 29	26					+								
30 to 32	35						+							
33	2						+	+						
34	1						+							
35 to 42	12							+						
43	65							+	+	+	+	+	+	+
44 to 45	3							+						
46	2							+		+				
47	2								+					
48	27								+	+		+		
49	6								+	+				
50	1								+					
51	4								+	+				
52 to 54	3								+					
55 to 57	4										+			
58 to 62	25													
63	10										+		+	
64–72	48										+			
73 to 80	13											+		
81 to 83	26												+	
	393													

The gray band indicates the Caete basin region plus Maranhao and Parnaiba estuaries; No =  Number of haplotypes. Shared haplotypes were included in the rectangle.

### Population structure

Results from AMOVA show that the genetic variation among regions (54.07%) was higher than the variation among populations within regions (17.43%). However, the variation within populations (28.5%) was larger than the variation among populations within regions (17.43%) (Table S1).

Pairwise Fst values between populations were all significant except for the comparisons between the Calcoene (1) vs the Amapa (2), Urindeua (7) vs Japerica (8) vs, and Caete (10) vs Sao Marcos (12) (Table 2). The highest pairwise Fst value (0.99309) resulted from comparisons between Calcoene (1) and Sao Marcos (12), while the lowest value (0.01339) resulted from comparisons between Urindeua (7) and Japerica (8).

**Table 2:** Fst results for a pairwise population comparison (lower diagonal) and associated significance indications (upper diagonal).

	1	2	3	4	5	6	7	8	9	10	11	12	13
1	0	ns	+	+	+	+	+	+	+	+	+	+	+
2	0.04195	0	+	+	+	+	+	+	+	+	+	+	+
3	0.97701	0.93216	0	+	+	+	+	+	+	+	+	+	+
4	0.96699	0.79903	0.8291	0	+	+	+	+	+	+	+	+	+
5	0.97337	0.91289	0.87763	0.63051	0	+	+	+	+	+	+	+	+
6	0.97686	0.84764	0.8767	0.64441	0.73499	0	+	+	+	+	+	+	+
7	0.96593	0.88517	0.96827	0.9510	0.96626	0.95477	0	ns	+	+	+	+	+
8	0.97646	0.88365	0.96847	0.95964	0.96901	0.96446	0.01339	0	+	+	+	+	+
9	0.21060	0.20455	0.78662	0.39189	0.68702	0.39965	0.39250	0.37186	0	+	+	+	+
10	0.97984	0.89587	0.97639	0.96801	0.97388	0.96957	0.18512	0.31842	0.33559	0	+	ns	+
11	0.97194	0.85115	0.96501	0.95626	0.96447	0.95962	0.38279	0.48772	0.19813	0.39686	0	+	+
12	0.99309	0.84830	0.96034	0.97215	0.96772	0.9769	0.10793	0.29739	0.24258	−0.02949	0.4189	0	+
13	0.75683	0.66764	0.77574	0.41010	0.68617	0.35274	0.61623	0.60177	0.22681	0.62504	0.51252	0.4936	0

Light and dark gray boxes indicate Fst values in the Para Platform and the Caete basin, respectively.

### Haplotype network

The haplotype network (Fig. S1) clearly distinguishes four major divisions identified as **Lineage 1** (the Amapa region), **Lineage 2** (the Amazon river mouth estuary), **Lineage 3** (the Para Platform) and **Lineage 4** (from Japerica to Parnaiba). The haplotype network showed that the Amazon estuary lineage (in blue) was slightly differentiated from the Para Platform population, but they are derived from a common ancestor. The populations from the Para Platform clearly show some degree of sub-structuring as a consequence of the relative isolation of populations from this region, a pattern demonstrated by a high number of unshared haplotypes. Conversely, the populations from Japerica to Parnaiba show a large number of shared haplotypes and a star-like pattern indicative of substantial gene flow. However, a surprising result was a close phylogenetic relationship of some haplotypes from Amapa and Calcoene (haplotypes 2, 4 and 6) with some haplotypes from the Caete estuary (haplotypes 69, 70, 71 and 72). As these geographic areas are 500 km apart and no evidence of present day connectivity is observed between these two regions, this certainly suggests a historical relationship between these two populations.

### Maximum likelihood analysis

**Figure 1:**
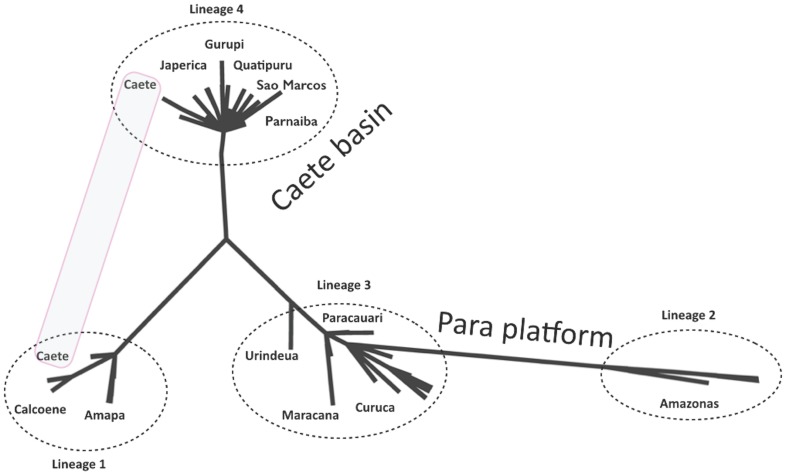
Maximum likelihood tree of the mitochondrial sequence of four-eyed fish from the North coast. This unrooted maximum-likelihood tree depicted the historical connection of haplotypes from the Caete and Amapa basins, which is indicated by a gray rectangle. The four lineages are delineated by interrupted line circles.

Maximum likelihood analysis produced the unrooted phylogenetic tree depicted in Fig. 1, which is very similar to the pattern showed by the haplotype network. The results clearly display the same four lineages described above. Again, the four haplotypes from the Caete estuary are more closely related to the haplotypes from Amapa and Calcoene than to any haplotype from the Caete estuary and neighboring estuaries.

### Demographic history

The results of Fu's Fs and R2 are presented in Table 3, including the significance of the simulated p-values. Fu's Fs and R2 were all negative but not significant for all populations from the Amapa coast (1 and 2) and Amazonas (3) as well as for three populations from the Para Platform (5–7), suggesting that these populations did not have an excess of rare nucleotide site variants compared to the expectation under a neutral model of evolution. Conversely, three populations in the Caete Basin (8, 9 and 11) showed negative and significant Fu's Fs or R2 estimates, indicating an excess of rare mutations when compared to the expected under the neutral model of evolution, which can be interpreted as a result of recent population expansion. Otherwise, these values can be explained by balancing selection on a nearby locus, although studies proving selection on the mitochondrial genome in natural populations are rare.

**Table 3:** Indexes of genetic variability and neutrality tests estimated in populations of *A. anableps* from 13 estuaries.

Collecting Places	Code	N	Hap	h	π	R2	Prob	Fu's Fs	Prob
Calcoene river estuary	1	23	3	0.170	0.00057	0.1492	0.3547	−1.30	0.06
Amapa river estuary	2	28	8	0.696	0.00460	0.1126	0.3967	−1.18	0.28
Amazonas river estuary	3	10	7	0.933	0.01065	0,1480	0.2939	−0.78	0.31
Paracauari river estuary	4	17	3	0.588	0.00250	0,1931	0.7843	1.44	0.80
Curuca Bay	5	26	10	0.775	0.00434	0.0687	0.0208	−3.78	0.01
Maracana river estuary	6	30	5	0.361	0.00140	0.0597	0.0001	−1.78	0.06
Urindeua Bay	7	30	14	0.903	0.01239	0.1635	0.9052	−2.22	0.19
Japerica river estuary	8	28	9	0.791	0.00352	0.0890	0.1298	−3.21	0.02
Quatipuru river estuary	9	22	8	0.701	0.00259	0.0810	0.0117	−3.95	0.005
Caete river estuary	10	98	17	0.788	0.01458	0.1164	0.8030	1.12	0.70
Gurupi river estuary	11	38	9	0.565	0.00154	0.0495	0.0008	−6.60	0.00
Sao Marcos Bay	12	30	5	0.501	0.00177	0.1039	0.2669	−1.16	0.20
Parnaiba river estuary	13	13	1	ne	ne	ne	ne	ne	ne
	Total	393	83	0.937					

N = number of individuals; Hap = number of haplotypes; h = haplotype diversity; π = nucleotidic diversity; Prob = probability.

**Figure 2:**
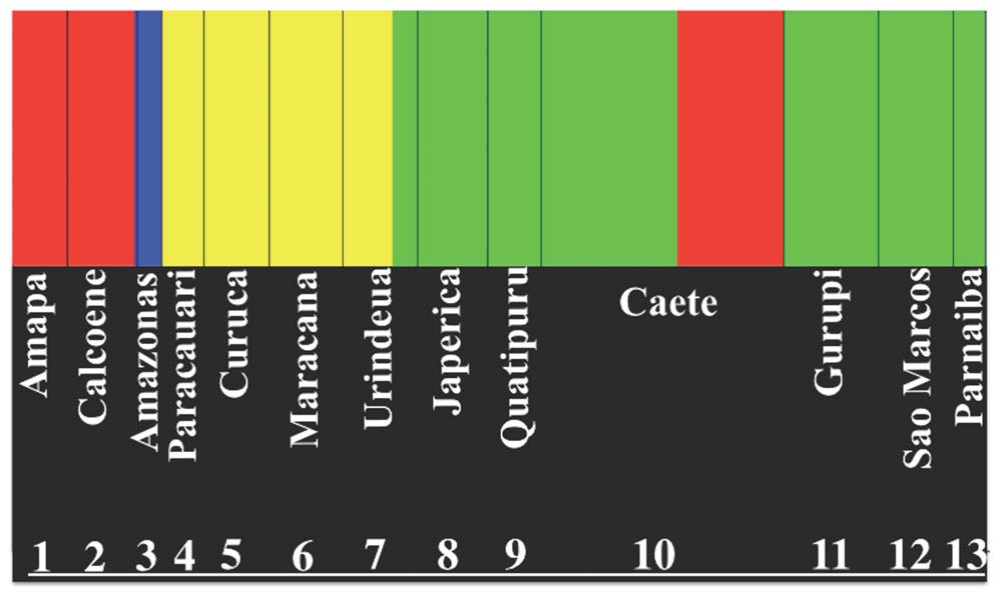
Bayesian analysis of population structure of the four-eyed fish from the North and Northeast coast. Locality and estuary names are shown. The clusters are indicated by colors: cluster 1 is red, cluster 2 is blue, cluster 3 is yellow, and cluster 4 is green. The haplotypes from Lineage 4, which are historically correlated with the Amapa basin, are represented in red inside cluster 4.

In perfect agreement with Fu's F and R2 results, the Bayesian Skyline Plot showed evidence of demographic expansion only in Lineage 4 (Japerica to Parnaiba), (see Fig. 2). However as divergence rates of control region are extremely variable in fishes the estimate obtained here (∼18,000 years ago) should be taken with caution due to uncertainty of divergence rates [23], [24]. Furthermore, there are evidences that estuaries were formed at about 5,000 years ago [3], therefore the inferred divergences should be much younger. Conversely, Lineages 1 (the Amapa coast) and 3 (the Para Platform) did not show any evidence of demographic expansion. Thus, it appears that these populations remained demographically stable during their entire existence. Lineage 2 (Amazon river mouth) was not tested because of its small size.

### Bayesian Analysis of Population Structure (BAPS)

**Figure 3:**
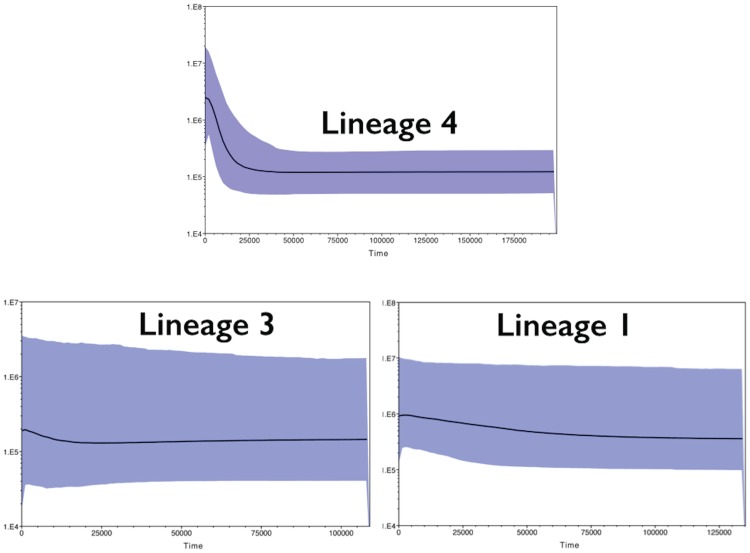
Bayesian Skyline Plot of three mitochondrial lineages of the four-eyed fish. Curves for each one of the three lineages are identified in the graph. The Y-axis indicates effective population size (Ne) x generation time, while the X-axis indicates mean time in thousands of years before present. The thick line represents the average, and the blue band represents the standard error.

Bayesian analysis of genetic structure identified four clusters (Fig. 3): Cluster I, all haplotypes from Calcoene and Amapa, one haplotype from Amazonas and four haplotypes from the Caete estuary; Cluster II, all remaining haplotypes from Amazonas; Cluster III, Paracauari, Curuça, Maracana, and Urindeua; Cluster IV, a few haplotypes from Urindeua plus Japerica, Quatipuru, Caete, Gurupi, Sao Marcos and Parnaiba. The four clusters correspond exactly to the same four lineages detected by the ML tree (Fig. 1).

## Discussion

### Phylogeographic Pattern & Past scenarios

The genetic analyses using the mtDNA Control Region revealed a phylogeographic pattern with four distinct mitochondrial lineages in populations of *A. anableps* from the North and Northeast coasts of Brazil.

Many studies with estuarine fishes suggest Pleistocene glacial events as the main cause of the genetic structuring [7]–[9], [25]–[27], and some studies with fresh water fishes [28], sponges [29], shrimps [30] and mollusks [31] ascribed the genetic structuring to Pleistocene vicariant events, with some in the beginning (1.806 million years before present) and others at the end of the Pleistocene (11,700 years before present). It is well known that, with the glacial advance during the Pleistocene, huge volumes of water were immobilized in continental ice sheets, resulting in short-term sea level drops of 100 meters or more over the entire surface of the Earth. At approximately 100,000 years ago, the sea level in the Amazon coast was approximately 130 m below the present day level [32]. The sea level oscillated during the last glaciation and reached its lowest level at the Last Glacial Maximum [33]. Under this circumstance, all the discharge of the Amazon river was most likely spread along the shelf, which means that the Amazon plume as it is today practically did not exist at that time (LGM). Moreover, the oceanic current regimes were quite different from what they are now. Therefore, it is quite possible that no physical (Amazon plume) or physicochemical (high salinity) barriers existed as they do now, allowing an ample dispersion of *Anableps* along the coast. Therefore, it is possible that allopatric refuges due to variations in sea level during the Pleistocene-Holocene associated with the biology of the species, such as viviparity and low potential for dispersal may have been the main force that promoted the recent divergence observed between populations and for the phylogeographic pattern observed for this species today. The divergence among lineages of the four-eyed-fish are comparable to those found in other species such as *Chelon haematocheilus*, a fish species from Asia [9], where three mitochondrial lineages were also detected that differentiated sometime between the middle and the end of the Pleistocene.

### Isolation by Distance

The Mantel test showed some evidence of isolation by distance when all populations where analyzed together (r = 0.45; P<0.01), but when each region was analyzed separately, no evidence of IBD was found. The structure observed in *A. anableps* can be explained by the geomorphological scenario of the study area, which was dramatically affected by sea level changes [3], [34]. Lineage 1 was isolated in the middle to upper portion of the Amapa coast, but curiously, Lineage 2 (the Amazonas river mouth) is more closely related to Lineage 3 (the Para Plataform) than to the Amapa Lineage 1, which is not consistent with the current flow of the Amazonas river to the north.

The geographic distribution of Lineages 2 (Amazonas), 3 (Para Platform) and 4 (Caete Basin to Parnaiba) matches the geomorphological landscape of the region. Therefore, it is highly probable that the wide distribution of tidal plains and the absence of interconnection between the estuaries have been essential for the isolation of Lineage 3 in the Para Platform. In contrast, there is an opposite pattern in the Caete Basin, which favored the demographic and spatial expansion of Lineage 4. Importantly, in contrast to the proposal of at least five geomorphological sectors [17], the entire region from the Caete estuary to the Parnaiba Delta seems to be a unique biogeographical region for populations of *A. anableps*. Therefore, the genetic data obtained here clearly distinguish the Para Platform and the Caete Basin but do not show differences between populations of the Caete Basin and those of Sao Marcos and Parnaiba. Thus, the genetic pattern of *Anableps* mitochondrial lineages helps to distinguish four biogeographical regions in the whole area of study: Oceanic Amapa (Calcoene and Amapa), the Amazonas river Mouth (near Macapa), the Para Platform (Paracauari, Curuça, Maracana, and Urindeua) and Caete-Parnaiba (Japerica, Caete, Quatipuru, Gurupi, Sao Marcos and the Paranaiba estuary).

The unexpectedly high genetic similarity between the four haplotypes from the Caete estuary and the haplotypes from Amapa (Lineage 1) may represent historical signatures of colonization of the Amapa estuaries by ancestral haplotypes from Caete that are widely distributed or are at least distributed from Caete to Amapa thousands of years ago, when the sea level was 130 m below the present level. Extinction of intermediate populations due to geomorphological changes, gradual increases in the sea level, formation of the Amazon plume, and oceanic currents may also have contributed. Lineage 1 is practically restricted to the Amapa State coast with no evidence of significant gene flow to neighboring populations, which is corroborated by the Bayesian Skyline Plot, suggesting that these populations did not experience a significant demographic expansion since colonization.

The genetic reconstruction performed here suggests that at least two distinct historical events helped shape the evolutionary history of *A. anableps* in northern Brazil, and both seem to be related to global events such as glaciations. The drop in the sea level during the Last Glacial period, which reached its lowest level (−130 meters) in Last Glacial Maximum (LGM; ∼22,000 to 19,000 years ago [33]), and the gradual rise to the current sea level (last 5,000 years [35]) drove the distribution and structuring of the populations as we know it today. Therefore, the exposure of the Atlantic shelf at the LGM period may have presented to the ancestral four-eyed-fish the opportunity for dispersion along the coast through a coastal lagoon environment [33] or even an Amazon “fresh water sea”. Second, and more recently, the rise of the sea level, the establishment of the Amazon plume, the configuration of the Marajo Archipelago [36], and the Oceanic Currents shaped the current distribution and genetic structuring of the *Anableps* populations analyzed here. It is interesting to note that the formation of the Marajó Archipelago during the recent Holocene separated the population of *Anableps* of the Amazonas river mouth from that of the Para Platform, from which the former is genetically derived.

Because the migration of four-eyed fish appears to be highly restricted to environments of low salinity [37], this suggests that the only possible connection between neighboring estuaries are by inland interconnections among the estuaries and not by the sea coast. Therefore, the absence of interconnection among the estuaries of the Para Platform provided the opportunity for the isolation of *Anableps* populations independent of distance. This isolation is clearly validated by the absence of shared haplotypes in this region and high values of pairwise Fst. With the exception of neighboring populations from the Maracana and Urindeua estuaries that share few haplotypes, the remaining populations from this sector show strong estuary fidelity. Bayesian Skyline Plot analysis (Fig. 2) also suggests that these populations did not experience a significant demographic expansion since colonization, possibly due to the relative isolation of each population due to their inaccessible estuaries. In contrast, for populations of the Caete Basin-Parnaiba, Bayesian Skyline plotting and the low Fst values confirm the significant gene flow among the populations in this sector and provide evidence for expansion and genetic structuring probable during late Pleistocene and early Holocene.

## Conclusions

The present study revealed high genetic divergence among populations of *Anableps* and uncovered four distinct mitochondrial lineages for *A. anableps* on the North and Northeast Amazon coast of Brazil. The study also suggested that the genetic structuring of these populations is correlated to events of the Holocene (11,700 to present).

The discrepancy of the gene flow pattern observed along the coast of Para fits well with the geomorphological features that are known as the Para Platform and the Caete Basin. The former has no interconnections between adjacent estuaries, while in the latter, these connections are evident. This analysis identifies only four biogeographic regions (Amapa, the Amazonas river Mouth, the Para Platform and Caete-Parnaiba) instead of the several geomorphological sectors described by geologists. The divergence observed in populations of *A. anableps* confirms that estuarine-resident organisms exhibit a degree of differentiation that is higher than strictly marine species [7], [8], [27]. Therefore, the results of the present work can be useful for designing new experiments to better understand the evolutionary history of the estuaries on the Brazilian coast using organisms that are biologically similar to the four-eyed-fish *Anableps*.

## Methods

### Ethics statement

Animal samples were collected and manipulated (DNA extraction, capture, transport and handling) under a permit (N° 12773-1) provided by the Environment Ministry (MMA) that was given to Iracilda Sampaio. All work was performed in compliance with and approved by the Ethics Committee of the University Federal do Para.

### Sampling

**Figure 4:**
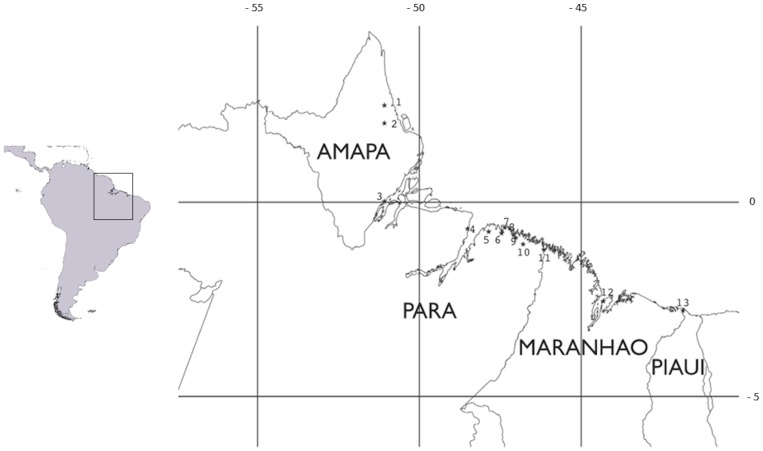
Map of the distribution of *Anableps anableps* populations sampled in the present study. Amapa, Para and Maranhao are states from North and Piaui from Northeast of Brazil. The estuaries are represented by the following numbers: 1 = Calcoene; 2 = Amapa; 3 = Amazonas; 4 = Paracauari; 5 = Curuca; 6 = Maracana; 7 = Urindeua; 8 = Japerica; 9 = Quatipuru; 10 = Caete; 11 = Gurupi; 12 = Sao Marcos; 13 = Parnaiba.

For the present study, samples of muscle tissue were obtained from a total of 393 specimens collected in 13 estuaries from four Brazilian states: Amapa, Para, Maranhão and Piaui, practically covering the entire range of the geographic distribution of the four-eyed fish in Brazil (Table 4; Fig. 4). Specimens were captured using fishnets and then euthanized by an overdose of tricaine methanosulphonate. After that a fragment of 5 to 10 mm of muscle tissue was collected from dorsal side of the fish. Muscle tissue samples were then fixed in absolute ethanol and later stored in a freezer at −20°C until DNA extraction.

**Table 4:** Origin, number of *A. Anableps* samples collected and geographic coordinates of the estuaries.

Collecting Sites	Code	Number	Longitude	Latitude
Amapa-Curuca-Amazonas				
Calcoene	1	23	−50.825245	2.500976
Amapa	2	28	−50.794464	2.051082
Amazonas	3	10	−51.104504	0.000842
Para Platform				
Paracauari	4	17	−48.534042	−0.717226
Curuça	5	26	−47.851547	−0.738175
Maracana	6	30	−47.462582	−0.765942
Urindeua	7	30	−47.346126	−0.631339
Caete Basin				
Japerica	8	28	−47.089771	−0.8276
Quatipuru	9	22	−47.005656	−0.899611
Caete	10	98	−46.678575	−0.980565
Gurupi	11	38	−46.139187	−1.221839
Sao Marcos estuary - Parnaiba Delta				
Sao Marcos	12	30	−44.551371	−2.532688
Parnaiba	13	13	−41.814806	−2.908765
Total		393		

### DNA extraction, PCR amplification and DNA sequencing

Total DNA was isolated from muscle tissue by digestion with proteinase K using a modified protocol with phenol chloroform and precipitation with sodium acetate and isopropanol [38]. A fragment of 464 base pairs of the Control Region (CR) was amplified by polymerase chain reaction (PCR) using the following primers: D-Loop L1 (5′-CCTAACTCCCAAAGCTAGGTATTC-3′) and D-Loop H1(5′-TGTTTATCACTGCTGAGAGTTCCCT-3′) [39]. Each PCR reaction consisted of 4 µl dNTP (1.25 mM), 2.5 µl buffer (10X), 1 µl MgCl2 (50 mM), 0.25 µl of each primer (100 ng/µl), 1 µl DNA (200 ng/µl), 0.2 µl Taq DNA polymerase (5 U/µl) and distilled water to complete the final reaction volume of 25 µl. The amplification program consisted of an initial step of 94 °C for 3 min (initial denaturation) followed by 35 cycles at 94°C for 30 seconds (denaturation), 57°C for 1 minute (annealing), and 72°C for 1 minute (extension). A final extension period of 7 minutes at 72°C was used. The amplified products were cleaned with EXOSAP-IT, and then, cycle sequencing was conducted using the Applied Biosystems Big Dye (ABI PrismTM Dye Terminator Cycle Sequencing Reading Reaction – PE Applied Biosystems – Carlsbad, CA-USA). The product was cleaned by precipitation and electrophoresed in an automatic capillary ABI 3130 (Applied Biosystems – Carlsbad, CA-USA) sequencer. The 393 DNA sequences were deposited in Genbank under the accession numbers from KJ639422 to KJ639814.

### Population analyses

The sequences obtained were aligned in the program CLUSTAL W [40] implemented in BioEdit [41] and edited manually. All statistical parameters and tests were estimated using Arlequin v. 3.5 [42]. Genetic variability within populations was estimated by computing the haplotype (h) and nucleotide diversities (π). Partitioning of genetic variation within and among populations was calculated using analysis of molecular variance (AMOVA) [42] by computing the conventional **F**-statistics from haplotypes with 1000 permutations using Arlequin v. 3.5 [42].

The optimal model of sequence evolution was determined using the Akaike Information Criterion with Kakusan v.4 [43]. The resulting model was the Hasegawa-Kishino-Yano (HKY) with among-site rate variation assuming a gamma distribution. Using this model in a script, generated by Kakusan v.4, a maximum-likelihood phylogenetic tree was reconstructed with PhyML version 3.0 [44]. From this tree, a haplotype genealogy was generated using Haploviewer 1.0 [45].

To test for past population expansion, two statistical tests were used: Fu's Fs, which uses the distribution of haplotypes (or alleles), and the R2 Statistic, which is based on the relationships between singletons and the average number of nucleotide differences. Fu' Fs and R2 statistics [46] proved to be very strong for this purpose, especially when population sample sizes are large (>50, Fu's Fs) or when sample sizes are small (<10, R2). Furthermore, the power of the R2 statistic is relatively high when the number of segregating sites is low (e.g., <20) [46]. The significance of Fu's Fs and R2 was obtained by examining the null distribution of 5,000 coalescent simulations of these statistics using DnaSP v.5 [47]. Significantly large negative Fu's Fs values and significantly positive R2 values were taken as evidence of a population expansion.

The reconstruction of genealogy and demographic history was made in a Bayesian framework using the Bayesian Skyline Plot implemented in Beast v1.7.5 [48]. As there is no fossil record to calibrate the time, the mutation rate was used to inform the prior distribution of the rate in the Bayesian analysis. The averaged divergence rate for the fish mtDNA control region used in the present work was 3.6×10^−8^ [49], which has been extensively used in fishes when the fossil record is absent.

Effective population sizes (*Ne*) against time were drawn for each one of the following lineages: 1 (Amapa, 51 individuals), 3 (the Para Platform, 103 individuals), and 4 (the Caete Basin, 229 individuals). Lineage 2 was not tested because it has only 10 individuals. The analyses were run in Beast v1.7.5 for 100 million iterations using default parameters and priors (HKY substitution model without site heterogeneity; strict molecular clock model; tree prior: coalescent Bayesian skyline, with 10 groups and piecewise-constant skyline model; operators: auto-optimize; log parameters: every 10,000 iterations). The results of the analyses were visualized using Tracer v1.5 [50]. Convergence of the chains to the stationary distribution was systematically confirmed by visual inspection of plotted posterior estimates.

The Mantel test was performed in Arlequin v. 3.5 [42] to check if the patterns of genetic differentiation found in *A. anableps* correspond to a model of isolation by distance (IBD). Four options were used: 1) all 13 populations; 2) Caete Basin; (3) Para Platform; and (4) Amapa Basin. The matrices used were pairwise *Fst* and geographic distances among populations.

To infer the number of subpopulations and to assign individual samples to these groups, we employed the BAPS6 software [51] a program for Bayesian inference of genetic structure in a population. When testing for population clusters, 10 replicates for every level of k (k is the maximum number of clusters) up to k = 13 were run. The number of reference individuals was set to 200, and admixture analyses were run 50 times per individual.

## Supporting Information

Figure S1 **Haplotype genealogy of the mitochondrial sequence based on a maximum-likelihood tree.** Circles represent haplotypes; the size is proportional to the number of individuals, and small blue dots represent intermediate, unsampled haplotypes. The four lineages are identified by distinct colors.
(TIFF)

Table S1 (DOC)
