# Supplementary figures and images for: The Past and Present of an Estuarine-Resident Fish, the “Four-Eyed Fish” Anableps anableps (Cyprinodontiformes, Anablepidae), Revealed by mtDNA Sequences
Source: PLoS One. 2014 Jul 8;9(7):e101727. doi: 10.1371/journal.pone.0101727 (PMC4086964; doi:10.1371/journal.pone.0101727)

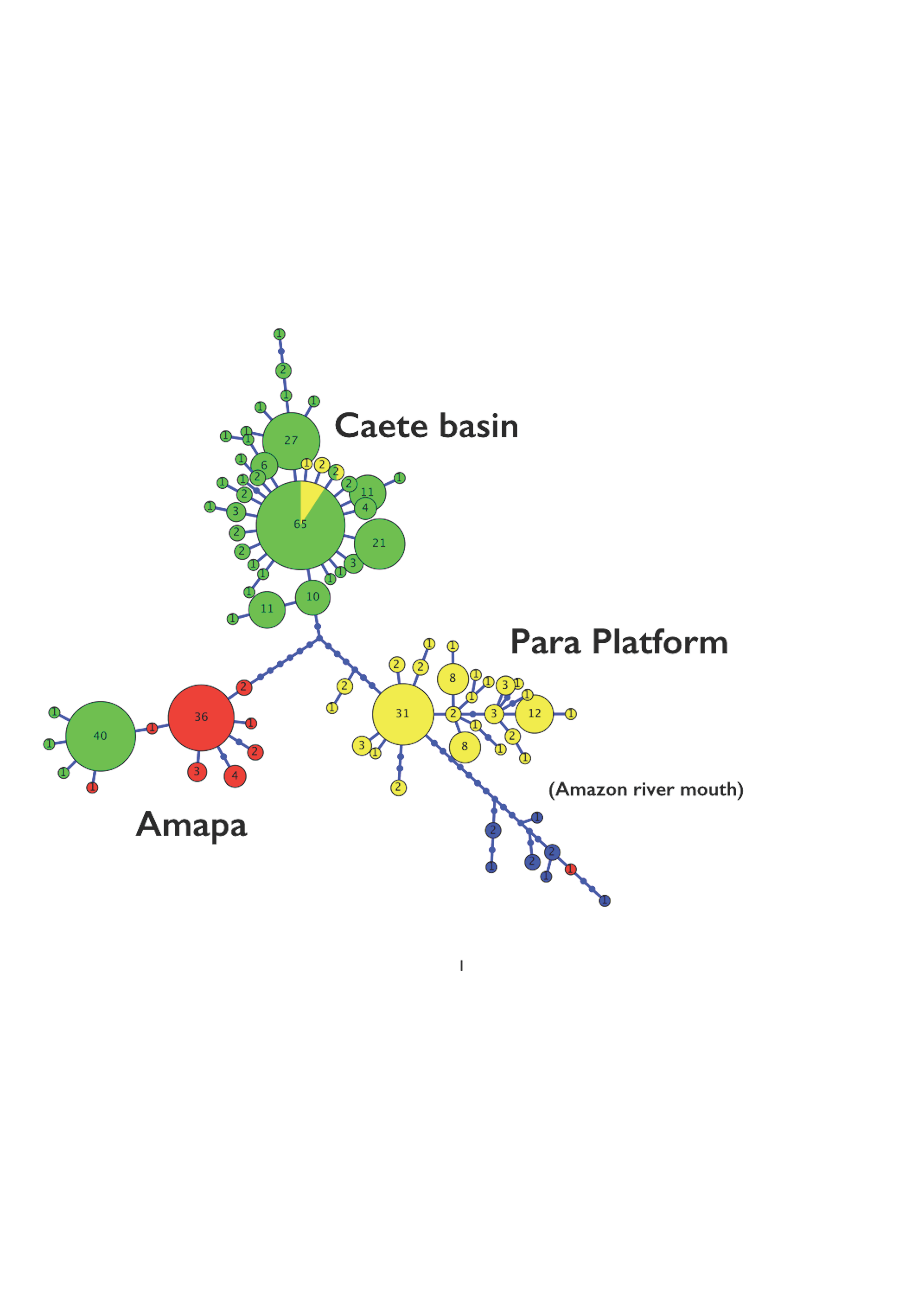

Supplement: Figure S1 — Haplotype genealogy of the mitochondrial sequence based on a maximum-likelihood tree. Circles represent haplotypes; the size is proportional to the number of individuals, and small blue dots represent intermediate, unsampled haplotypes. The four lineages are identified by distinct colors. (TIFF) [file pone.0101727.s001.tif]
