# Supplementary material for: The Past and Present of an Estuarine-Resident Fish, the “Four-Eyed Fish” Anableps anableps (Cyprinodontiformes, Anablepidae), Revealed by mtDNA Sequences
Source: PLoS One. 2014 Jul 8;9(7):e101727. doi: 10.1371/journal.pone.0101727 (PMC4086964; doi:10.1371/journal.pone.0101727)
Supplement: Table S1 — (DOC) [file pone.0101727.s002.doc]

## Table 1S - Hierarchical analysis of the molecular variance (AMOVA) of mtDNA control region haplotypes of *A. anableps*

| Source of variation | Degree of freedom | Sum of squares | Variance components | F-statistics | Total (%) | P-value |
| --- | --- | --- | --- | --- | --- | --- |
| Among groups | 3 | 702.5 | 2.79 | FCT=0.540 | 54.07 | <0.001 |
| Among populations within groups | 9 | 257.6 | 0.89 | FSC=0.339 | 17.43 | <0.001 |
| Within populations | 380 | 558.6 | 1.47 | FST=0.715 | 28.50 | <0.001 |
| Total | 392 | 1518.7 | 5.16 | | | |
